# Biodistribution and Physiologically-Based Pharmacokinetic Modeling of Gold Nanoparticles in Mice with Interspecies Extrapolation

**DOI:** 10.3390/pharmaceutics11040179

**Published:** 2019-04-12

**Authors:** Mohamed Aborig, Paul R. V. Malik, Shruti Nambiar, Pierre Chelle, Johnson Darko, Anthony Mutsaers, Andrea N. Edginton, Andre Fleck, Ernest Osei, Shawn Wettig

**Affiliations:** 1 School of Pharmacy, University of Waterloo, Kitchener, ON N2G 1C5, Canada; maborig@uwaterloo.ca (M.A.); prvmalik@uwaterloo.ca (P.R.V.M.); snambiar@uwaterloo.ca (S.N.); pierre.chelle@uwaterloo.ca (P.C.); wettig@uwaterloo.ca (S.W.); 2 Grand River Regional Cancer Centre, Grand River Hospital, Kitchener, ON N2G 1G3, Canada; johnson.darko@grhosp.on.ca (J.D.); andre.fleck@grhosp.on.ca (A.F.); ernest.osei@grhosp.on.ca (E.O.); 3 Department of Physics and Astronomy, University of Waterloo, Waterloo, ON N2L 3G1, Canada; 4 Department of Clinical Studies, Ontario Veterinary College, University of Guelph, Guelph, ON N1G 2W1, Canada; mutsaers@uoguelph.ca; 5 Department of Systems Design Engineering, University of Waterloo, Waterloo, ON N2L 3G1, Canada

**Keywords:** nanoparticles, pharmacokinetics, biodistribution

## Abstract

Gold nanoparticles (AuNPs) are a focus of growing medical research applications due to their unique chemical, electrical and optical properties. Because of uncertain toxicity, “green” synthesis methods are emerging, using plant extracts to improve biological and environmental compatibility. Here we explore the biodistribution of green AuNPs in mice and prepare a physiologically-based pharmacokinetic (PBPK) model to guide interspecies extrapolation. Monodisperse AuNPs were synthesized and capped with epigallocatechin gallate (EGCG) and curcumin. 64 CD-1 mice received the AuNPs by intraperitoneal injection. To assess biodistribution, groups of six mice were sacrificed at 1, 7, 14, 28 and 56 days, and their organs were analyzed for gold content using inductively coupled plasma mass spectrometry (ICP-MS). A physiologically-based pharmacokinetic (PBPK) model was developed to describe the biodistribution data in mice. To assess the potential for interspecies extrapolation, organism-specific parameters in the model were adapted to represent rats, and the rat PBPK model was subsequently evaluated with PK data for citrate-capped AuNPs from literature. The liver and spleen displayed strong uptake, and the PBPK model suggested that extravasation and phagocytosis were key drivers. Organ predictions following interspecies extrapolation were successful for rats receiving citrate-capped AuNPs. This work lays the foundation for the pre-clinical extrapolation of the pharmacokinetics of AuNPs from mice to larger species.

## 1. Introduction

Nanotechnology engineering can be defined as the engineering of matter at the nanoscale. The use of nano-sized structures can unlock many unique properties, such as higher surface energy, greater thermal and electrical conductivity, easier penetration through biological barriers and surface plasmon resonance [1,2]. Many important biological structures and interactions occur at the nanoscale, and researchers have harnessed this knowledge to develop nanoparticles (NPs) that can influence cellular and molecular structures in biological systems [3,4,5]. One of the most promising nanomaterials being researched for biomedical applications is the gold nanoparticle (AuNP). The potential benefits of AuNPs are well-recognized, with applications in drug delivery, medical imaging, bio-sensing and radiation enhancement. 

AuNPs have many attractive properties for biomedical applications such as biocompatibility, low toxicity, surface functionalization, small dimensions and high X-ray attenuation [6]. 

There are many ways to synthesize AuNPs, but few procedures use non-toxic and environmentally benign biological methods. The “green” synthesis of metallic NPs uses plant extracts as the reducing and stabilizing agents. The plant extracts used for synthesizing AuNPs in this study are epigallocatechin gallate (EGCG) and curcumin. Both curcumin and EGCG are nutraceuticals, and are heavily researched for their potential to act as antioxidants for preventing DNA damage [7]. EGCG is a type of catechin found in green-tea extract, and curcumin is a principal curcuminoid found in turmeric. The abilities of plant extracts to reduce metal ions to NPs are due to the inherent antioxidant properties of these natural molecules. Many plants are rich in antioxidant molecules, which can act as electron-donating agents in a redox reaction [8]. The administration route chosen for this study was intraperitoneal injection. This method was chosen due to its simplicity for large scale animal studies and higher tolerance for injection volumes [9]. One of the first biomedical applications of AuNPs was pursued in 1971, when AuNPs were first used for immunogold labeling in electron microscopy [10]. However, since that time there have been very few successful translations of AuNPs to humans as a therapeutic agent [11]. Part of this challenge is due to uncertainty about the biodistribution, pharmacokinetics and toxicity of AuNPs. 

It is important to consider the pharmacokinetics (PK) of NPs during pre-clinical development for biomedical applications, in order to ascertain the likelihood of achieving sufficient exposure in tissues of interest, while minimizing systemic toxicity. In contrast to traditional small molecule drugs, most NPs exhibit complex PK in vivo due to a variety of physicochemical and biological factors. The uncertainties in these factors limit pre-clinical assessments, especially when extrapolating PK between routes of administration, or between species (e.g., mouse to rat or monkey to human) [12]. For example, uptake into cells of the mononuclear phagocyte system is fast and variable between particle sizes, shapes and charges, and also between species, [12,13,14,15,16], complicating predictions of tissue exposure. In addition, large particle size and poor permeability across the capillary endothelium often limit the ability of NPs to enter interstitial spaces in organs, particularly in organs with continuous or tight capillary junctions (e.g., brain, muscle, skin) [17,18]. Instead, they often accumulate in organs with sinusoidal or open capillary junctions (e.g., liver, spleen, tumors) [12,17]. 

Physiologically-based pharmacokinetic (PBPK) models offer promising opportunities to quantify these factors, and guide PK assessment in pre-clinical development. There are three main components of PBPK models:

1. Anatomy and physiology of the individual
2. Physicochemical properties of the drug
3. System of equations governing the movement and interaction of the drug within the body (model processes).

PBPK models offer distinct advantages over top-down or “data-driven” models for extrapolation, because when one component is altered for the extrapolation scenario, the confidence gained in the original scenario is conserved, increasing confidence in the final predictions. For example, extrapolation of PK between species is possible by altering the anatomical and physiological parameters in the model to represent the desired species, while holding the mathematical description of the drug and the model processes constant. 

Significant growth in the development of PBPK models for NPs has occurred in the past decade [14,17,19]. However, they lag behind the field of macromolecular drug modeling, which has advanced rapidly with the popularity of monoclonal antibody drugs and plasma factor concentrates, among other products [18,20,21]. Here we incorporate components from macromolecular models into the framework of NP PBPK modeling, replacing tissue-specific partition coefficients with a physiologic approach to extravasation and transcytosis, adding lymph recirculation and hepatocyte uptake, and proposing a global approach to phagocytosis by cells of the mononuclear phagocyte system. 

Overall, we integrate experimental techniques with an in silico approach to the biodistribution of AuNPs in mice to maximize learning, and set the stage for interspecies extrapolation in the pre-clinical development of AuNPs as a drug product.

## 2. Materials and Methods 

### 2.1. Materials

Epigallocatechin gallate (≤95% purity, E4143–Aldrich, St. Louis, MO, USA), curcumin (≥98% purity, Alexis Biochemicals, San Diego, CA, USA) and Gold (III) chloride trihydrate (HAuCl_4_·3H_2_O, 520918-Aldrich) were purchased for the synthesis of Gold nanoparticles (AuNPs), capped with epigallocatechin gallate (EGCG). 1000 ppm (µg/mL) gold standard for concentration measurements was purchased from Inorganic Ventures and Sigma-Aldrich (38168–Aldrich, St. Louis, MO, USA).

### 2.2. Synthesis and Isolation 

The synthesis protocols for EGCG-AuNP and Curc-AuNP were adapted from a protocol by Nambiar et al. [22]. The first step required solubilizing the polyphenol compound, which was accomplished by adding 10 mM NaOH to a vial containing EGCG or curcumin powder to form a 1 mM solution. The solution was then held in an ultrasonic bath (44 °C) and 200 µL of 100 mM HAuCl_4_·3H_2_O was added dropwise. The formation of EGCG-AuNP/Curc-AuNP was confirmed by the sudden change of the color of the solution to a ruby-red. The final step of the synthesis was to remove unreacted EGCG/curcumin and HAuCl_4_ in the solution through dialysis. The solution was transferred from the vial to a 3.5 kDa dialysis tubing (SnakeSkin Dialysis Tubing, Thermo Fisher, Waltham, MA, USA) that was then placed in a 2 L beaker of MilliQ water for ≥24 h to allow the unreacted molecules to diffuse out of the solution. The beaker was placed in an ice-cooler with a stir bar and stir plate to allow stirring of the dialysis medium. The composition of the final solution includes poly-phenol coated AuNPs dispersed in water.

### 2.3. Characterization 

#### 2.3.1. Ultraviolet Visible (UV-Vis) Absorption Spectroscopy 

UV-Vis absorption measurements were carried out with SpectraMax Molecular Devices M5 plate reader (San Jose, CA, USA) immediately after synthesis, to confirm the formation of AuNPs. A single strong absorbance band between 500–600 nm is an indicator of the formation of AuNPs. 

#### 2.3.2. Dynamic Light Scattering (DLS) 

The hydrodynamic sizes, particle size distributions and zeta potentials of EGCG-AuNPs and Curc-AuNPs were measured with light-scattering methods using the Malvern Zetasizer Nano-ZS (Malvern Instruments Ltd., Malvern, UK). The hydrodynamic size of EGCG-AuNP or Curc-AuNP includes the Au core, EGCG/curcumin coating, and solvent layer. The hydrodynamic radius is a key input into the physiologically-based pharmacokinetic (PBPK) model, as will be discussed.

#### 2.3.3. Transmission Electron Microscopy (TEM)

The size and shape of AuNPs were investigated with TEM. A few small drops of EGCG-AuNP or Curc-AuNP solution were drop-casted on a 200-mesh Formvar copper grid, and TEM images were obtained with a Philips CM10 electron microscope at 60 kV. TEM size measurements display only the size of the Au core, and exclude the outer coating of EGCG or curcumin.

### 2.4. Animal Study 

A total of 122 CD-1 mice were housed at the animal research facility of the University of Waterloo (Waterloo, ON, Canada). All procedures involving mice were approved by the office of research ethics at the University of Waterloo (AUPP Number 17-14). 30 mice were administered EGCG-AuNPs by intraperitoneal (IP) injection at 10 mg of Au per kg of body weight (average injection volume = 371.04 µL), another 34 mice were administered Curc-AuNPs by intraperitoneal injection at 10 mg of Au per kg of body weight (average injection volume = 377.93 µL) and 58 more mice were used as control subjects, and received 500 µL saline by IP injection (Table 1). The weight of each mouse was recorded every three days. During weighing sessions, the mice were examined, and any changes in behavior or physical abnormalities were recorded. 

### 2.5. Gold Quantification

ICP-MS (Teledyne Leeman Labs) is a mass spectroscopy technique used for accurately determining the Au concentration of the synthesized EGCG-AuNPs and Curc-AuNPs. The Au content was determined based on a calibration curve of five HAuCl_4_ standards, which were 200 µg/mL, 400 µg/mL, 600 µg/mL, 800 µg/mL and 1000 µg/mL. It was also used to quantify the concentration of Au in the digested mouse organs. The impacts of the biological media on the method detection limit (MDL) and limit of quantification (or quantitation—LOQ) of ICP-MS measurements were assumed negligible. Mice were sacrificed at 1, 7, 14, 28 and 56 days, and their organs were acid-digested and analyzed for Au content. The extracted organs were liver, spleen, lung, stomach, kidney and heart. The brain was not selected for analysis, because AuNP accumulation was expected to be low or negligible. Large particle size, negative surface charge and poor lipophilicity hinder the ability of EGCG-AuNPs and Curc-AuNPs to cross the blood–brain barrier. In addition, opsonization and adherence of plasma proteins can further increase particle size, and hinder particle penetration into the brain [23,24].

CO_2_ inhalation was used to euthanize the mice. The CO_2_ flow rate was approximately 2 L/min, and CO_2_ flow was maintained in the cage for 10 min. The mice were dissected, and tissues of the listed organs were extracted and placed in 50 mL polystyrene vials. All instruments and samples were thoroughly washed with water to minimize cross-contamination of AuNPs between samples. The samples were weighed using a Metler Toledo AG245 Dual balance. This step was followed by an addition of 1.5 mL aqua regia (HNO_3_ + 3HCl) and 8.5 mL of MilliQ H_2_O to each vial, and placement upon a heat block to facilitate digestion. Each vial was left on the heat block for at least three hours, and the temperature on the heat block was set at 175 °C. The vials were then filtered using a vacuum filter pump with DigiFilter 0.45 micron tubes. The filtered liquid in the vials was labeled and sent to the University of Guelph (located in Guelph, Ontario, Canada) analytical laboratory for precise quantification of Au content using ICP-MS. 

The calibration curve for quantifying Au content in the organs was generated using a blank, and five standard products (1 to 25 parts per billion). Calibration curve correlation coefficients were all >0.995. The calibration curve was verified with a standard prepared using a different source of Au, and acceptable when within ±10% of the expected value. The method detection limit (MDL) for Au in solution was 3 ppb, and the limit of quantitation (LOQ) for Au in solution provided was 10 ppb. The instrument analysis was performed on ^197^Au, while ^195^Pt and ^205^Pt were used as internal standards to correct for varying plasma conditions.

### 2.6. PBPK Modelling Workflow

**Figure 1:**
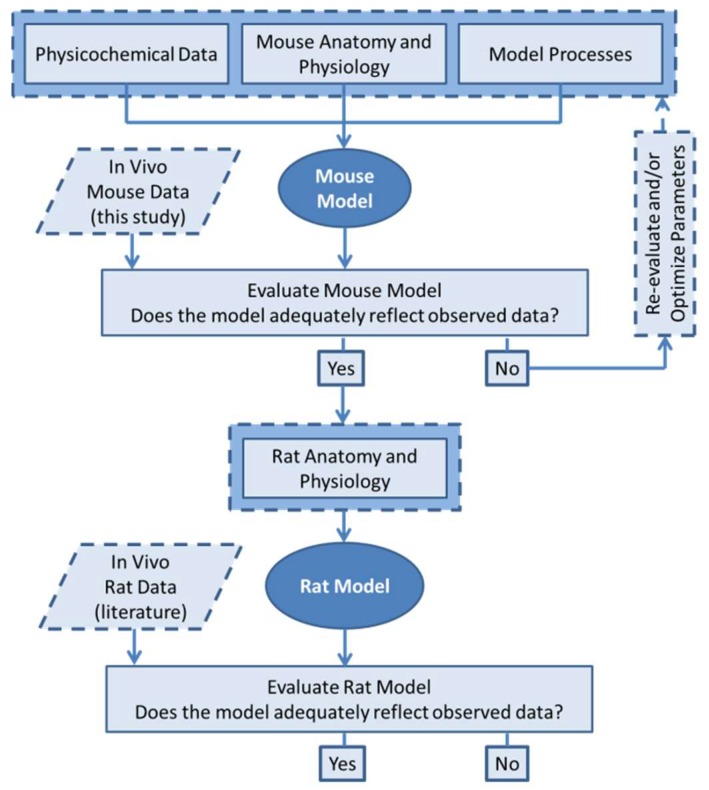
Physiologically-based pharmacokinetic (PBPK) modeling workflow.

Biodistribution data was collected for both EGCG-AuNPs and Curc-AuNPs, and used to inform the physiologically-based pharmacokinetic (PBPK) model for mice, which was then extrapolated to rat species using literature data [25,26]. Due to their similar physicochemical characteristics and biodistribution data, the two particles were considered equivalent entities in the model. The modeling workflow in Figure 1 was used to develop the PBPK model for nanoparticles (NPs) in mice using data from this study, and evaluate its utility for extrapolation to other species.

### 2.7. Software

The PBPK model was built and evaluated in MATLAB R2017a using the Intiquan toolbox (IQMTools v1.2.2.2) by Henning Schmidt. Experimental data from the literature was digitized using the PlotDigitizer v2.6.8 application by Joseph Huwaldt. 

### 2.8. Model Structure

The whole-body PBPK model structure for large molecules and therapeutic proteins by Niederalt et al. [18] was adapted with features from recent NP PBPK models [14,19,27,28]. Within the virtual body, fifteen organs were represented as mathematical compartments, and connected by plasma and lymph flows. The fifteen organs modeled were the heart, kidneys, muscle, skin, brain, adipose, gonads, liver, stomach, spleen, pancreas, small intestine, large intestine, bone and lungs. Each organ was divided into four sub-compartments: plasma, vascular endothelium, macrophages and interstitial space. 

**Figure 2:**
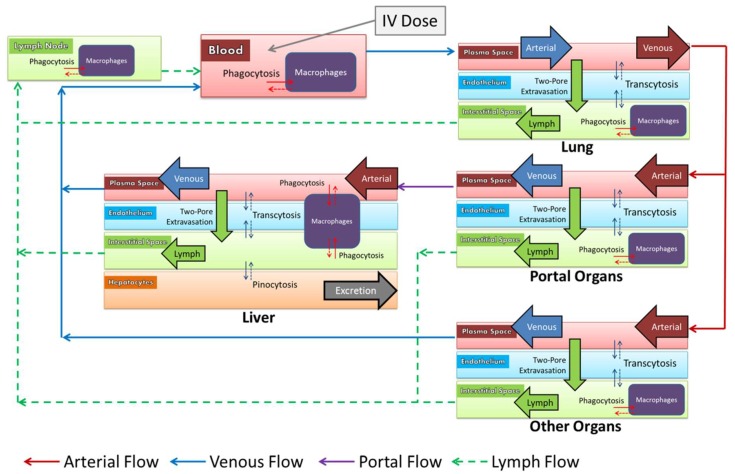
Nanoparticles (NPs) circulate throughout the body, as pictured. They may cross into the interstitial spaces of organs by extravasation or transcytosis across the endothelium. Phagocytic uptake into macrophages and pinocytic uptake into hepatocytes are key determinants of biodistribution. Lymph flow recycles NPs from the interstitial space back into the venous blood. Particles are excreted from hepatocytes into the bile. Renal excretion is negligible for particles >10 nm in diameter.

The model processes are described in Figure 2. Within organs, NPs in the plasma sub-compartment moved across the endothelium into the interstitial space by extravasation or transcytosis. The interplay of convection and diffusion as it relates to extravasation was described by the two-pore model, which accounts for the hydrodynamic radius of the particle, and the dynamic fluid flux across large and small pores in the capillary wall [18,29]. Transcytosis was mediated by pinocytosis into the vascular endothelium, and exocytosis to the opposite side. 

In the interstitial space, NPs were taken up into macrophages by phagocytosis. Fluid in the interstitial space drained into the lymphatic system, which recycled NPs back to the venous blood via the thoracic duct. In the liver, macrophages known as Kupffer cells lined the sinusoids and phagocytozed NPs directly from the plasma. A unique sub-compartment in the liver described pinocytosis into hepatocytes, and subsequent excretion into the bile. Renal excretion was assumed negligible for NPs larger than 10 nm in diameter [30]. Intraperitoneal (IP) administration was described as a bolus into an administration compartment from which NPs diffused into the interstitial spaces and into lymph nodes surrounding the portal organs (stomach, spleen, pancreas, small intestine and large intestine). The model equations are presented in the Supplementary Materials.

### 2.9. Model Parameterization

#### 2.9.1. Anatomy

Organ volumes, compositions, plasma flows and lymph flows for a 28 g mouse and a 280 g rat were obtained from PK-Sim (www.open-systems-pharmacology.org). Lymph node volumes were obtained from Shah and Betts [31]. 

#### 2.9.2. Extravasation

The two-pore model for extravasation was implemented as outlined by Niederalt et al. [18]. A lymph reflection coefficient $\left(\sigma^{IS}\right)$ described the resistance to lymph flow in the interstitial space due to particle size and physical and electrostatic interactions with the extracellular matrix [31].

#### 2.9.3. Vascular Endothelium

The endothelial fraction of each organ (*i*) was derived from vascular surface area by:

$$F_{i}^{E}=k_{e}\times F_{i}^{V}\times d_{e}$$

where $k_{e}$ is a constant of proportionality (950 cm^2^/mL), $F_{i}^{V}$ is the vascular fraction of the organ, and $d_{e}$ is the thickness of vascular endothelium (3 × 10^−5^ cm) [18]. These parameters are assumed species-independent in the absence of additional physiologic data. The rate of pinocytosis into the vascular endothelium (CLup) was set to 0.05 μL/h/μL endothelial cells in agreement with the majority of PBPK models for large molecules to date [31,32,33]. The rate of exocytosis was assumed to be equal to the rate of pinocytosis to maintain fluid balance in endothelial cells.

#### 2.9.4. Macrophages

Macrophage volume in each organ $\left(V_{i}^{M}\right)$ was calculated as a fraction of the cellular volume. The following factors were used as reference values to assign macrophage fractions to other organs:

1. Kupffer cells and stellate macrophages make up 10% of the cellular volume of the liver [34,35]
2. Complete blood counts indicate that 1% of the blood volume is comprised of phagocytic cells, based on an average white blood cell volume of 1.25 pL [36]
3. White pulp macrophages make up 30% of the cellular volume of the spleen [37,38,39]

To quantify macrophages in other organs by these reference mediums, RNA expression data for 7 proteins that are largely specific to macrophage populations (CD14, CD40, CD11b, EMR1, CD68, CSF-1 and CSF-1R) and the macrophage volumes listed in two PBPK models were compiled [19,33]. Based on these factors, the remaining organs were categorized as having prominent (4%) or low (2%) macrophage content. The macrophage fractions for all organs are listed in the Supplementary Materials.

The maximum rate of phagocytosis (Pup) is drug-specific and required optimization in the absence of any in vitro data. A range between 0.075 and 40 μL/h/μL macrophages was reported in the PBPK study by Lin et al. [27], and these boundaries were used for the optimization. Exocytosis in macrophages was assumed to be the same as in endothelial cells (0.05 μL/h/μL macrophages).

Phagocytosis of NPs is a saturable process. Therefore, a Michaelis-Menten approximation was implemented for the phagocytosis rate in each organ (*i*):

$$Pup_{i}=Pup\times V_{i}^{M}\times \left(1-\frac{C_{i}^{M}}{K_{M}+C_{i}^{M}}\right)$$

where $C_{i}^{M}$ is the concentration of AuNPs in the macrophages, and $K_{M}$ is the half-saturation concentration in the cells (5000 μg/mL) based on the maximum NP capacity reported by Alkilany and Murphy [40].

In the liver, macrophages were present lining the sinusoidal capillaries. The fraction of phagocytic uptake $\left(F_{up}^{M}\right)$ from plasma and the fraction of exocytosis back to plasma $\left(F_{rec}^{M}\right)$ were both set to 0.5 in the absence of any physiologic data. This ratio may represent the ratio of Kupffer cell volume to stellate macrophage volume in the liver.

#### 2.9.5. Hepatocytes

Hepatocyte volume in the liver $\left(V_{Liver}^{H}\right)$ was calculated as 75% of the cellular volume [35]. The rates of pinocytosis and exocytosis in hepatocytes were assumed to be the same as the respective rates in the vascular endothelium. The rate of NP excretion into bile $\left(K_{Bile}\right)$ is drug-specific, and required optimization.

#### 2.9.6. Intraperitoneal Administration

**Figure 3:**
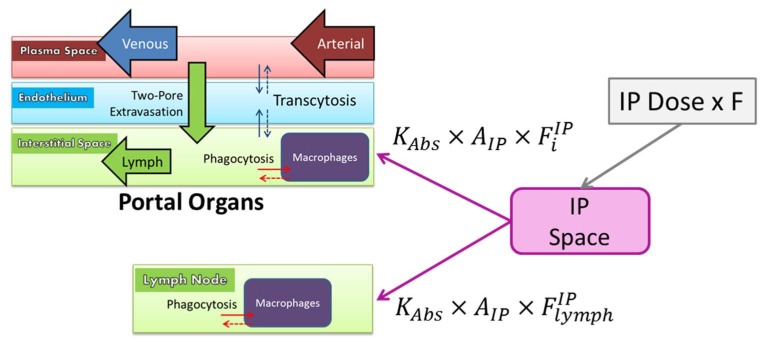
Schematic depicting the intraperitoneal (IP) injection route of administration. A fraction of NPs diffuses from the IP space into the interstitial spaces of the portal organs and lymph node before reaching systemic circulation.

AuNPs diffused into the interstitial spaces of the portal organs (stomach, spleen, pancreas, small intestine and large intestine) and into the lymph, according to a first order rate constant ($K_{Abs}$) (Figure 3). An optimized bioavailability (F) was applied to the dose, based on the observation that a portion of the dose remained trapped in the intraperitoneal space upon dissection. 

The fraction of the bioavailable dose that diffused into the interstitial space of each portal organ (*i*) was proportional to the respective organ volume, while 10% was absorbed directly into the lymph.

$$F_{i}^{IP}=\left(1-F\right)\times 0.9\times \frac{V_{i}}{V_{portal}};\;F_{lymph}^{IP}=\left(1-F\right)\times 0.1$$

#### 2.9.7. Optimization

The maximum rate of phagocytosis (Pup), the rate of excretion into bile $\left(K_{Bile}\right)$, the lymph reflection coefficient $\left(\sigma^{IS}\right)$ and the IP bioavailability (F) were optimized to the experimental data in mice for the liver and spleen obtained from this study. Appropriate identifiability of the four parameters was confirmed prior to the optimization by assessing local sensitivity and parameter correlation with the *IQMidentifiability* function. A simulated annealing temperature-based algorithm was used for the optimization.

### 2.10. Model Evaluation

The utility of the PBPK model for extrapolating biodistribution to other species was evaluated with two experimental datasets for non-PEGylated AuNPs in rats from literature (Table 2) [25,26]. Anatomical and physiological parameters in the mouse model were adapted to represent a mean rat with a weight of 280 g (see above), while all other parameters and processes in the model were unchanged. The rat model was simulated, and the absolute average fold error across all data points was calculated to compare model predictions to observed values for each available organ in the literature datasets possessing measurements significantly different from controls.

### 2.11. Sensitivity Analysis

A local sensitivity analysis was performed to capture how each parameter may affect NP exposure in the liver and spleen. For the analysis, each parameter was varied independently by ±10% of the initial value, and the resulting percent changes in the liver and spleen area-under-the-curve (AUC) outputs were reported.

## 3. Results

### 3.1. Characterization

#### 3.1.1. Ultraviolet Visible (UV-Vis) Absorption Spectroscopy 

**Figure 4:**
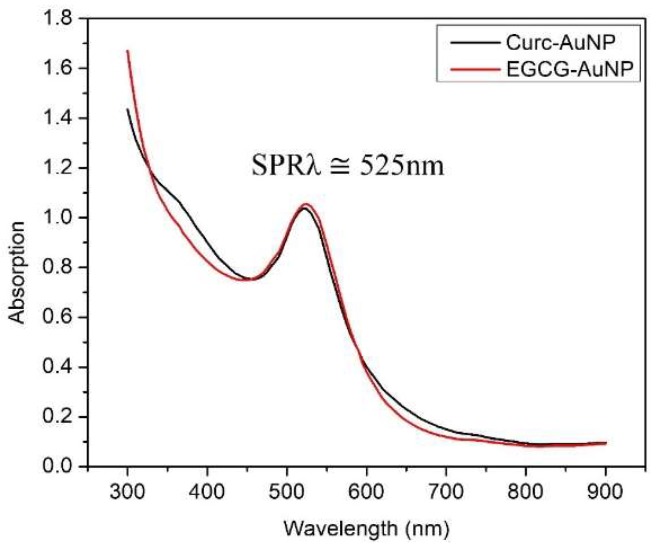
Ultraviolet Visible (UV-Vis) absorption at 525 nm signifies reduction of HAuCl_4_ salt by the nutraceutical and formation of Gold nanoparticles (AuNPs). The peaks for Curc-AuNPs and epigallocatechin gallate (EGCG)-AuNPs are almost identical, and therefore we can expect both groups of NPs to have similar shape and size.

UV-Vis measurements were taken immediately after synthesis and the surface plasmon resonance peak was found at 525 nm (SPRλ ≅ 525 nm) (Figure 4). 

#### 3.1.2. Dynamic Light Scattering (DLS) 

Once the EGCG-AuNP or Curc-AuNP solution was dialyzed, DLS measurements were taken (Table 3). The average hydrodynamic diameter for EGCG-AuNPs was 25.00 nm with poly-dispersity index (PDI) of 0.173, and zeta potential of -22 mV. The average hydrodynamic diameter for Curc-AuNPs was 19.62 nm, with polydispersity index (PDI) of 0.167 and zeta-potential of −20 mV.

#### 3.1.3. Transmission Electron Microscopy (TEM)

**Figure 5:**
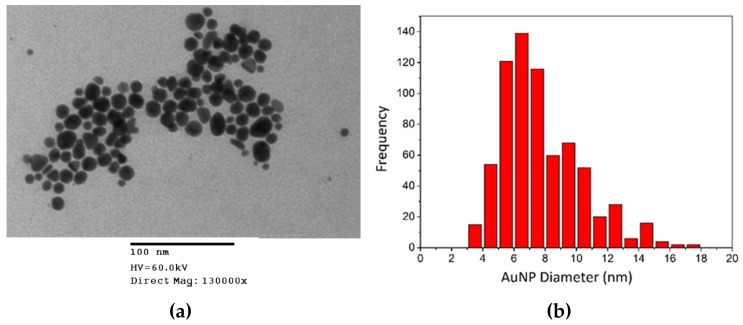
(**a**) TEM image of EGCG-AuNPs. The dark areas represent the AuNP cores where electron attenuation is high. (**b**) A histogram of Au core diameters of EGCG-AuNPs.

The size and shape of the AuNPs were studied using TEM. Figure 5 depicts a TEM image of EGCG-AuNPs with variable shape. The main shape is spherical, though there are triangular and hexagonal AuNPs observed in the image. The EGCG surface coating is not visible in TEM images, because it is an organic molecule with low electron attenuation. 

### 3.2. Animal Study 

The concentrations of the EGCG-AuNPs and Curc-AuNPs from synthesis were 422.77 µg/mL and 391.43 µg/mL, respectively. However, the advised upper limit for IP injection volume was 0.5 mL, and the desired dose (10 mg Au/kg) would require a larger injection volume. In order to meet this requirement, the AuNP solutions were further concentrated by centrifugation to increase the Au concentration to 700 µg/mL. This resulted in an average injection volume of 0.38 mL per mouse. A single IP injection of the AuNPs was administered 4–5 mm deep into the peritoneal cavity for all. There were 122 mice used in the study. All mice were weighed every three days in the morning, and no significant changes in body weight or food consumption were observed.

### 3.3. Gold Quantification

**Figure 6:**
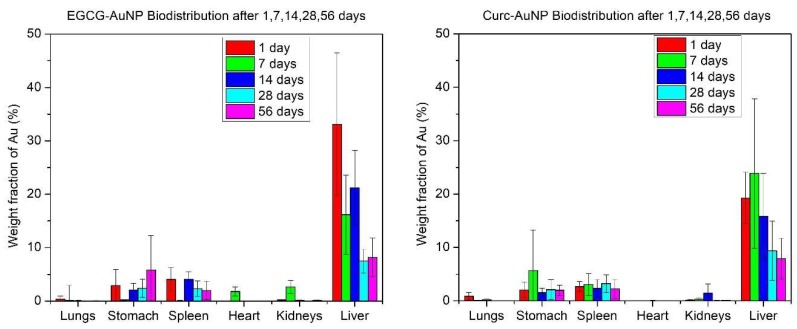
Weight fractions of injected AuNP dose found in select organs after acid digestion.

Elemental Au was detected in most organs. The highest amounts were found in the liver and spleen. The distribution pattern is shown in Figure 6. The average accumulated Au amounts in the liver, expressed as percentages of injected dose for 1, 7, 14, 28, 56 days, were 33.11%, 16.18%, 21.23%, 7.49%, and 8.18% for EGCG-AuNP and 19.28%, 23.88%, 15.85%, 9.38%, and 7.92% for Curc-AuNPs.

### 3.4. Model ParameterizationOptimization

#### Optimization

**Figure 7:**
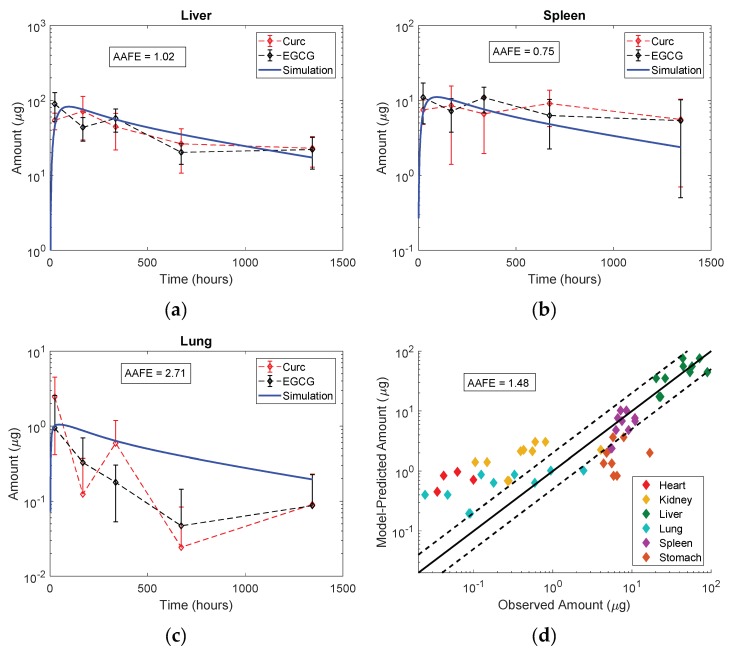
Final mouse model simulations compared to the observed biodistribution data for organs in this study: (**a**) liver (**b**) spleen and (**c**) lung. (**d**) shows the model-predicted vs. observed amounts in each organ with relation to the equality line achieving an absolute average fold error of 1.48.

The optimized values for the maximum rate of phagocytosis (Pup), the rate of excretion into bile $\left(K_{Bile}\right)$, the lymph reflection coefficient $\left(\sigma^{IS}\right)$ and the IP bioavailability (F) were 0.995 mL/h/mL macrophages, 0.0128 h^−1^, 0.64 and 0.76 respectively. Figure 7 shows the final mouse model simulations compared to the observed biodistribution data for Curc- and EGCG-capped AuNPs in this study. Despite the wide variability in the data, an absolute average fold error of 1.48 was achieved between the model predictions and the observed mean values in all organs. All organ profiles after fitting to liver and spleen are available in the Supplementary Materials. While the overall fit was acceptable, the model tended to over-predict amounts in organs with very low or near-zero exposure (heart, kidney and lung) and under-predict amounts in portal organs (spleen and stomach) after IP administration. The optimized model was then evaluated.

### 3.5. Model Evaluation

**Figure 8:**
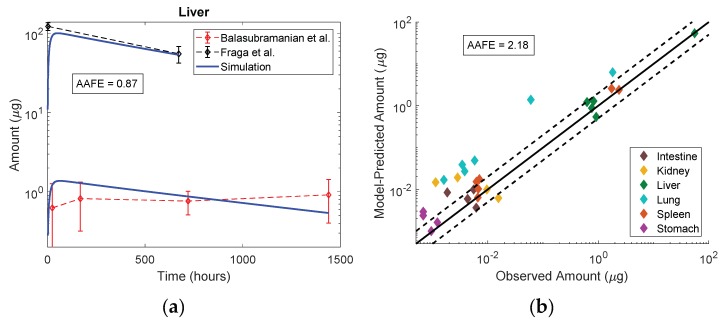
Rat model simulations compared to the observed biodistribution data in rats after IV administration from Balasubramanian et al. [25] and Fraga et al. [26] (**a**) shows the liver profiles and (**b**) shows the model-predicted vs. observed amounts in each organ with relation to the equality line achieving an absolute average fold error of 2.18.

The mouse model was successfully adapted to represent rats with anatomical and physiological parameters. Figure 8 demonstrates the utility of the PBPK model for extrapolation across particle sizes, doses (0.01, 0.7 and 10 mg Au/kg), routes of administration (IP vs. IV) and species (mouse vs. rat). Liver profiles were approximated well at all doses. As with the original mouse model, there was a trend for an over-prediction in organs with very low exposures. Given the uncertainty in the data and the heterogeneity in the experiments, the model was proficient for rats with predictions across all organs achieving an absolute average fold error of 2.18. Much of the error was driven by an over-prediction of the Au content in the lung. All organ profiles for model evaluation are available in the Supplementary Materials.

### 3.6. Sensitivity Analysis

**Figure 9:**
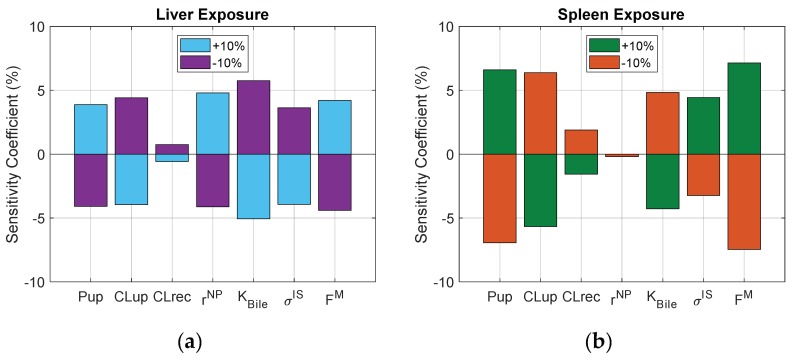
Sensitivity of liver (**a**) and spleen (**b**) exposures to relative perturbations in key parameters. Pup = maximum rate of phagocytosis, CLup = rate of pinocytosis, CLrec = rate of exocytosis, $r^{NP}$ = NP hydrodynamic radius, $K_{Bile}$ = rate of excretion into bile, $\sigma^{IS}$ = lymph reflection coefficient and $F^{M}$ = macrophage fraction of organ.

To assess the relative importance of each process in the biodistribution of NPs after IV administration in mice, key parameters were perturbed by ±10%, and the resulting changes in liver and spleen AUC outputs are reported in Figure 9. When comparing the two, liver exposure is more sensitive to changes in extravasation (e.g., solute radius) and spleen exposure is more sensitive to changes in cellular uptake. 

## 4. Discussion

The green synthesis of EGCG-AuNPs and Curc-AuNPs was simple, inexpensive, free of toxic reagents, and produced monodisperse NPs with a nutraceutical coating. The synthesis procedure is reproducible, and provides NPs of a controlled size and shape. Figure 4 illustrates the SPR peaks at 525 nm for both particles, suggesting spherical and monodisperse morphology. The strong overlap of SPR peaks suggests both EGCG-AuNP and Curc-AuNP have similar physical characteristics. This observation was corroborated by DLS measurements and TEM images (Table 3). The negative zeta potentials associated with the nutraceutical caps helped to stabilize the AuNPs through electrostatic repulsion [41]. Colloidal stability may decrease at high pH, but the nutraceutical coating can provide some steric stability in these conditions [42]. The stability of AuNPs in solution is a crucial property because its biodistribution, movement through pores and cellular uptake rates are directly influenced by its ability to stay dispersed. 

During the animal study there were no adverse effects reported for any of the mice treated with either EGCG-AuNPs or Curc-AuNPs. All mice exhibited normal weight progression with no significant weight loss. After consolidation of the biodistribution data from the IP study, the majority of AuNPs accumulated in the liver and spleen (Figure 6). The Au amounts in the organs, particularly in the liver, decreased for mice that were part of the longer duration studies, which the PBPK model attributed to lymph drainage from the organs. The liver is a common organ for large molecules and toxins to accumulate and be cleared from the body via excretion into feces. Many sources in literature have shown AuNP biodistribution to be greatly affected by size [43,44,45]. According to an IV study by Hirn et al. in rats, 95–98% of 18, 80 and 200 nm AuNPs accumulated in the liver after 24 h, while 51.3% and 81.6% of 1.4 nm and 2.8 nm AuNPs accumulated in the liver after 24 h, respectively [43]. 

The PBPK model was designed and calibrated with the biodistribution data generated in this mouse study by IP administration of 10 mg/kg EGCG- and Curc-AuNPs. Following optimization to the liver and spleen profiles for these entities, the model demonstrated convincing accuracy for describing Au amounts in each organ over time (liver, spleen, heart, kidney, lung and stomach), with an absolute average fold error of 1.48 (Figure 7). The model over-predicted Au amounts in organs with very low or near zero exposure. While the absolute difference is negligible, this phenomenon may be explained by the loss of blood or other organ fluid during sample preparation and transfer, or misspecification of convection and diffusion parameters in the two-pore model, as it has not been validated for very large particles. There were notable uncertainties in the modeling of IP administration. High observed amounts in the stomach are not traditionally seen following IV administration of inorganic NPs, and thus the under-prediction of the model for this organ was attributed to this uncertainty. 

The test for interspecies extrapolation to rats passed with reasonable accuracy, achieving an absolute average fold error of 2.18 (Figure 8). While most organs were within 2.5-fold error there was a trend for over-prediction of Au amounts in organs with very low or near-zero exposure, especially the lung (AAFE = 9.10 across the data from both studies). The lung organ composition is unique because it is made up of 80% extracellular fluid. If the over-prediction error is due to loss of organ fluid (blood, interstitial fluid) during sample obtainment and transfer, then it follows logically that the lung presents with the highest error. 

The overall accuracy in interspecies extrapolation is acceptable given the extrapolation not only across species, but also across doses, routes of administration, particle sizes and experimental designs. While the calibration data in this study was generated at a dose of 10 mg/kg IP in mice, the evaluation datasets were generated at doses of 0.01 and 0.07 mg/kg by IV administration in rats with particles of significantly larger hydrodynamic radii (median 12 nm in calibration vs. 23 nm in evaluation datasets), and using citrate as the alternative capping agent. Citrate maintains a similar negative charge to curcumin and EGCG, and in the absence of any in vitro studies comparing the various capping agents, the impact of this physicochemical alteration on biodistribution was not accounted for in the model. Further exploration of the impact of surface charge on phagocytic uptake may inform these features in the future.

Several variations of PBPK models for pharmaceutical NPs have emerged in the last decade [17]. The model proposed herein is an amalgamation of these efforts that has been supplemented with additional features from protein-based drug models, such as two-pore extravasation, lymphatic recycling and hepatocyte uptake. In addition, it is the first PBPK model in scientific literature to consider the IP administration of large molecules. The focus in development was to use parameters that can be informed from in vitro experiments, such as particle radii and cellular uptake rates. It may be near impossible to predict the impact of NP charge and surface coating on biodistribution. However, by running simple in vitro tests, researchers can assess the impact of charge or surface coating modifications on cellular uptake rates, and input that information into the PBPK model. This combined in vitro/in vivo/in silico approach is essential to generating confidence in pre-clinical development models.

While this work lays a framework for modeling the biodistribution of inorganic NPs, there are additional challenges and opportunities for expansion. Serum proteins have been shown to adhere to the surface of inorganic NPs according to charge-based interactions forming a “biocorona,” which may dictate the rates and routes of cellular uptake [46,47,48]. As examples, receptor-mediated endocytosis may be achieved when proteins of the complement cascade are activated and phagocytosis may be triggered when opsonins adsorb to the NP surface. Formation of the biocorona occurs over time, and may occur differently in different species depending on the nature of serum proteins that are present, and the circulation time of the particle in the bloodstream [46,47,48]. PBPK models can be used to describe the latter phenomenon mechanistically, and reduce uncertainty in interspecies extrapolation. 

A handful of limitations must be acknowledged. The heterogeneity in experimental design, small sample sizes and large variability in PK results limit precise assessments of model accuracy in silico, instead giving a general picture of particle disposition in vivo. Blood data was not collected prior to animal sacrifice, though this information would thoughtfully improve the understanding of absorption to the bloodstream after IP administration, and the rates of distribution across the vascular endothelium. As such, the rates of extravasation and transcytosis appear to underestimate the rates of organ uptake after IV administration in the evaluation dataset. Of course, the doses in the evaluation datasets are up to 1000-fold lower by weight, and we cannot rule out dose-dependent organ uptake. The two-pore model has not been formally evaluated with particles larger than 10 nm in radius, and this may be an example of the minor overestimation of convection and diffusion for this class of particles. Finally, while median particle size was an input into the PBPK model, the distribution of particle sizes in the administered solution was not considered mathematically. This assumption is in part justified by the low polydispersity index of the final formulations (<0.2).

## 5. Conclusions

In conclusion, we have demonstrated the utility in silico modelling to supplement experimental findings from in vitro and in vivo studies, increasing understanding and improving efficiency in the pre-clinical development of large molecule drug products. This work lays the foundation for the extrapolation of the pharmacokinetics of AuNPs from mice to larger species.

## Figures and Tables

**Table 1:** Number of mice sacrificed for each time point and study group.

Time Point (Days)	Number of Mice in EGCG-AuNP Arm	Number of Mice in Curc-AuNP Arm
1	5C, 5T (10 total)	5C, 6T (11 total)
7	5C, 5T (10 total)	5C, 6T (11 total)
14	5C, 5T (10 total)	5C, 6T (11 total)
28	7C, 8T (15 total)	7C, 8T (15 total)
56	7C, 7T (14 total)	7C, 8T (15 total)

C represents control, T represents treatment.

**Table 2:** Experimental datasets from literature used for model evaluation.

Dataset	Species	Particle ^a^	Dose	Organ PK ^b^	Meas. Time
Balasubramanian et al. [25]	Rat	20 nm Au with citrate	0.01 mg Au/kg IV	Liver, Spleen, Lung, Kidney, Stomach, Intestine	1, 7, 30 and 60 days
Fraga et al. [26]	Rat	16 nm Au with citrate	0.7 mg Au/kg IV	Liver, Spleen, Lung	30 min and 28 days

^a^ Particle size is reported as the diameter measured by Transmission Electron Microscopy (TEM) visualization. ^b^ Pharmacokinetic (PK) data was extracted for organs with non-zero values that were different from controls, and converted to amounts in organs (μg).

**Table 3:** Characterization data of EGCG-AuNPs and Curc-AuNPs.

AuNP	Hydrodynamic Diameter (nm)	Polydispersity Index (PDI)	Zeta Potential (mV)	Au Conc. (µg/mL)	Au Core Diameter (nm)	Nanoparticle Concentration (AuNP/mL)
EGCG-AuNP	25.00 ± 0.172	0.173 ± 0.007 ^a^	−22	422.790	7.78 ± 2.59	$8.1\times 10^{13}$
Curc-AuNP	19.62 ± 0.09	0.167 ± 0.002	−20	391.430	6.21 ± 3.36	$1.6\times 10^{14}$

^a^ ± represents the standard deviation of the reported mean value.

## Supplementary Materials

Supplementary Materials are available online at https://www.mdpi.com/1999-4923/11/4/179/s1. Table S1. State, Variable and Parameter Notation, Table S2. Anatomical Values for Mouse, Table S3. Organ Volumes and Composition for Mouse, Table S4. Fluid Flow Rates for Mouse, Table S5. Extravasation Parameters, Table S6. Macrophage Content, Table S7. Model Constants, Table S8. Anatomical Values for Rat, Table S9. Organ Volumes and Composition for Rat, Table S10. Fluid Flow Rates for Rat, Figure S1: Comparison of Model Simulations to Mouse Biodistribution Data from this Study, Figure S2: Comparison of Model Simulations to Rat Biodistribution Data from Balasubramanian et al., Figure S3: Comparison of Model Simulations to Rat Biodistribution Data from Fraga et al.
