# Supplementary material for: Biodistribution and Physiologically-Based Pharmacokinetic Modeling of Gold Nanoparticles in Mice with Interspecies Extrapolation
Source: Pharmaceutics. 2019 Apr 12;11(4):179. doi: 10.3390/pharmaceutics11040179 (PMC6523871; doi:10.3390/pharmaceutics11040179)
Supplement: Supplementary file 1 [file pharmaceutics-11-00179-s001.pdf]

pharmaceutics

Supplementary Materials: Biodistribution and Physiologically-Based Pharmacokinetic Modeling of Gold Nanoparticles in Mice with Interspecies Extrapolation

Mohamed Aborig, Paul R.V. Malik, Shruti Nambiar, Pierre Chelle, Johnson Darko, Anthony Mutsaers, Andrea N. Edginton, Andre Fleck, Ernest Osei, Shawn Wettig

Model Equations

Table S1. State, Variable and Parameter Notation.

Term	Units	Definition
PLQ_{Lung}	mL/h	Total body plasma flow
$L_{LymphNode}$	mL/h	Total body lymph flow
PLQ_i	mL/h	Plasma flow into and out of each organ
L_i	mL/h	Lymph flow into and out of each organ
C_{Plasma}	µg/mL	Concentration of NP in plasma
$C_{LymphNode}$	µg/mL	Concentration of NP in lymph
C_i^P	µg/mL	Concentration of NP in organ plasma space
C_i^E	µg/mL	Concentration of NP in organ endothelium
C_i^M	µg/mL	Concentration of NP in organ macrophages
C_i^{IS}	µg/mL	Concentration of NP in organ interstitial space
C_{Liver}^H	µg/mL	Concentration of NP in liver hepatocytes
F_i^V	-	Vascular space fraction (plasma and blood)
F_i^P	-	Plasma space fraction
F_i^E	-	Endothelium fraction
F_i^M	-	Fraction of cellular volume attributed to macrophages
F_i^{IS}	-	Interstitial fraction
F_i^C	-	Cellular fraction
F_{Liver}^H	-	Fraction of cellular volume attributed to hepatocytes
V_{Plasma}	mL	Volume of plasma
$V_{LymphNode}$	mL	Volume of lymphatic system components
V_i	mL	Volume of organ
$V_{i_{mouse}}$	mL	Volume of organ in a 21g mouse
V_i^P	mL	Volume of organ plasma space
V_i^E	mL	Volume of organ endothelium
V_i^M	mL	Volume of organ macrophages
V_i^{IS}	mL	Volume of organ interstitial space
V_{Liver}^H	mL	Volume of liver hepatocytes
Pup	mL/h/mL	Rate of phagocytosis per mL of macrophages
K_M	µg/mL	Michaelis-Menten constant for saturable phagocytosis
F_{up}^M	-	Fraction of phagocytic uptake from liver plasma
F_{rec}^M	-	Fraction of exocytosis from liver macrophages into plasma
$CLup$	mL/h/mL	Rate of pinocytosis and exocytosis per mL of endothelium, macrophages and hepatocytes

F_{up}^{E}	-	Fraction of pinocytic uptake from organ plasma space
F_{rec}^{E}	-	Fraction of exocytosis into organ plasma space
K_{Bile}	1/h	Rate of excretion into bile from liver hepatocytes
A_{IP}	μg	Amount of NP in the intraperitoneal space
K_{Abs}	1/h	Rate of absorption from the intraperitoneal space
F	-	Intraperitoneal bioavailability
F_{Portal}^{IP}	-	Fraction absorbed into the portal system
J_i^L	mL/h	Fluid flux across large pores
J_i^S	mL/h	Fluid flux across small pores
$Jiso_i$	mL/h	Fluid recirculation flow rate
P_i^L	cm/h	Permeability across large pores
P_i^S	cm/h	Permeability across small pores
Pe_i^L	-	Peclet number for large pores
Pe_i^S	-	Peclet number for small pores
S_i	cm^2	Surface area of vascular endothelium
γ_i^L	-	Ratio of NP hydrodynamic radius to organ large pore radius
γ_i^S	-	Ratio of NP hydrodynamic radius to organ small pore radius
r_i^L	nm	Organ large pore radius
r_i^S	nm	Organ small pore radius
r_i^{NP}	nm	NP hydrodynamic radius
α_i^L	-	Fraction of flow via large pores in organ
α_i^S	-	Fraction of flow via small pores in organ
K_{IP}	-	Interstitial:Plasma partition coefficient
Lp_i	mL/h/N	Hydraulic conductivity in organ
RT	N·cm/mol	Gas constant at body temperature (37°C)
N_a	1/mol	Avogadro's number
k_e	cm^2/mL	Proportionality constant for vascular endothelium
d_e	cm	Thickness of vascular endothelium
F_i^{Jiso}	-	Fluid recirculation fraction
F_i^{lymph}	-	Lymph flow fraction

Blood Compartment: Plasma

Equation S1: Amount of NP in Plasma

$$V_{Plasma} \times \frac{dC_{Plasma}}{dt}$$

$$= (PLQ_{Heart} - L_{Heart}) \times C^{P}_{Heart} + (PLQ_{Kidney} - L_{Kidney}) \times C^{P}_{Kidney}$$

$$+ (PLQ_{Muscle} - L_{Muscle}) \times C^{P}_{Muscle} + (PLQ_{Skin} - L_{Skin}) \times C^{P}_{Skin}$$

$$+ (PLQ_{Brain} - L_{Brain}) \times C^{P}_{Brain} + (PLQ_{Adipose} - L_{Adipose}) \times C^{P}_{Adipose}$$

$$+ (PLQ_{Gonads} - L_{Gonads}) \times C^{P}_{Gonads}$$

$$+ (PLQ_{Liver} - L_{Liver} + PLQ_{Stomach} - L_{Stomach} + PLQ_{Spleen} - L_{Spleen}$$

$$+ PLQ_{Pancreas} - L_{Pancreas} + PLQ_{SInt} - L_{SInt} + PLQ_{LInt} - L_{LInt}) \times C^{P}_{Liver}$$

$$+ (PLQ_{Bone} - L_{Bone}) \times C^{P}_{Bone} + L_{LymphNode} \times C_{LymphNode}$$

$$- \; PLQ_{Lung} \times C_{Plasma} - Pup \times V^{M}_{Plasma} \times \left(1 - \frac{C^{M}_{Plasma}}{K_M + C^{M}_{Plasma}}\right) \times C_{Plasma}$$

$$+ CLup \times V^{M}_{Plasma} \times C^{M}_{Plasma}$$

Equation S2: Concentration of NP in Plasma Macrophages

$$\frac{dC^{M}_{Plasma}}{dt} = Pup \times \left(1 - \frac{C^{M}_{Plasma}}{K_M + C^{M}_{Plasma}}\right) \times C_{Plasma} - CLup \times C^{M}_{Plasma}$$

Lymph Node Compartment

Equation S3: Amount of NP in Lymphatic System

$$V_{LymphNode} \times \frac{dC_{LymphNode}}{dt}$$

$$= K_{Abs} \times A_{IP} \times (1 - F_{Portal}^{IP}) + (1 - \sigma_{Heart}^{IS}) \times L_{Heart} \times C_{Heart}^{IS}$$

$$+ \left(1 - \sigma_{Kidney}^{IS}\right) \times L_{Kidney} \times C_{Kidney}^{IS} + (1 - \sigma_{Muscle}^{IS}) \times L_{Muscle} \times C_{Muscle}^{IS}$$

$$+ \left(1 - \sigma_{Skin}^{IS}\right) \times L_{Skin} \times C_{Skin}^{IS} + \left(1 - \sigma_{Brain}^{IS}\right) \times L_{Brain} \times C_{Brain}^{IS}$$

$$+ \left(1 - \sigma_{Adipose}^{IS}\right) \times L_{Adipose} \times C_{Adipose}^{IS}$$

$$+ (1 - \sigma_{Gonads}^{IS}) \times L_{Gonads} \times C_{Gonads}^{IS} + \left(1 - \sigma_{Liver}^{IS}\right) \times L_{Liver} \times C_{Liver}^{IS}$$

$$+ (1 - \sigma_{Stomach}^{IS}) \times L_{Stomach} \times C_{Stomach}^{IS}$$

$$+ \left(1 - \sigma_{Spleen}^{IS}\right) \times L_{Spleen} \times C_{Spleen}^{IS}$$

$$+ (1 - \sigma_{Pancreas}^{IS}) \times L_{Pancreas} \times C_{Pancreas}^{IS} + (1 - \sigma_{SInt}^{IS}) \times L_{SInt} \times C_{SInt}^{IS}$$

$$+ (1 - \sigma_{LInt}^{IS}) \times L_{LInt} \times C_{LInt}^{IS} + (1 - \sigma_{Bone}^{IS}) \times L_{Bone} \times C_{Bone}^{IS}$$

$$+ \left(1 - \sigma_{Lung}^{IS}\right) \times L_{Lung} \times C_{Lung}^{IS} - L_{LymphNode} \times C_{LymphNode}$$

$$- Pup \times V_{LymphNode}^{M} \times \left(1 - \frac{C_{LymphNode}^{M}}{K_M + C_{LymphNode}^{M}}\right) \times C_{LymphNode}$$

$$+ CLup \times V_{LymphNode}^{M} \times C_{LymphNode}^{M}$$

Equation S4: Concentration of NP in Lymphatic System Macrophages

$$\frac{dC_{LymphNode}^{M}}{dt} = Pup \times \left(1 - \frac{C_{LymphNode}^{M}}{K_M + C_{LymphNode}^{M}}\right) \times C_{LymphNode} - CLup \times C_{LymphNode}^{M}$$

Organ Sub-Compartment: Plasma Space

Equation S5: Amount of NP in Plasma Space of Organs (i = all organs except the Lungs and Liver)

$$V_i^P \times \frac{dC_i^P}{dt} = PLQ_i \times C_{Lung}^P - (PLQ_i - L_i) \times C_i^P$$

$$- \left(J_i^L \times (1 - \sigma_i^L) \times C_i^P + P_i^L \times S_i \times \left(C_i^P - \frac{C_i^{IS}}{K_{IP}} \right) \times \frac{Pe_i^L}{e^{Pe_i^L} - 1} \right.$$

$$\left. + J_i^S \times \left(1 - \sigma_i^S\right) \times C_i^P + P_i^S \times S_i \times \left(C_i^P - \frac{C_i^{IS}}{K_{IP}} \right) \times \frac{Pe_i^S}{e^{Pe_i^S} - 1} \right)$$

$$- CLup \times V_i^E \times C_i^P \times F_{up}^E + CLup \times V_i^E \times C_i^E \times F_{rec}^E$$

Equation S6: Amount of NP in Plasma Space of Lung (i = Lung)

$$V_i^P \times \frac{dC_i^P}{dt} = PLQ_i \times C_{Plasma}^P - (PLQ_i - L_i) \times C_i^P$$

$$- \left(J_i^L \times (1 - \sigma_i^L) \times C_i^P + P_i^L \times S_i \times \left(C_i^P - \frac{C_i^{IS}}{K_{IP}} \right) \times \frac{Pe_i^L}{e^{Pe_i^L} - 1} \right.$$

$$\left. + J_i^S \times \left(1 - \sigma_i^S\right) \times C_i^P + P_i^S \times S_i \times \left(C_i^P - \frac{C_i^{IS}}{K_{IP}} \right) \times \frac{Pe_i^S}{e^{Pe_i^S} - 1} \right)$$

$$- CLup \times V_i^E \times C_i^P \times F_{up}^E + CLup \times V_i^E \times C_i^E \times F_{rec}^E$$

Equation S7: Amount of NP in Plasma Space of Liver (i = Liver)

$$V_i^P \times \frac{dC_i^P}{dt} = PLQ_i \times C_{Lung}^P + (PLQ_{Stomach} - L_{Stomach}) \times C_{Stomach}^P$$

$$+ \left(PLQ_{Spleen} - L_{Spleen}\right) \times C_{Spleen}^P$$

$$+ (PLQ_{Pancreas} - L_{Pancreas}) \times C_{Pancreas}^P + (PLQ_{SInt} - L_{SInt}) \times C_{SInt}^P$$

$$+ (PLQ_{LInt} - L_{Lint}) \times C_{LInt}^P$$

$$- \ \big(PLQ_i - L_i + PLQ_{Stomach} - L_{Stomach} + PLQ_{Spleen} - L_{Spleen}$$

$$+ PLQ_{Pancreas} - L_{Pancreas} + PLQ_{SInt} - L_{SInt} + PLQ_{LInt} - L_{LInt}\big) \times C_i^P$$

$$- \left(J_i^L \times (1 - \sigma_i^L) \times C_i^P + P_i^L \times S_i \times \left(C_i^P - \frac{C_i^{IS}}{K_{IP}}\right) \times \frac{Pe_i^L}{e^{Pe_i^L} - 1}\right.$$

$$\left. + J_i^S \times \left(1 - \sigma_i^S\right) \times C_i^P + P_i^S \times S_i \times \left(C_i^P - \frac{C_i^{IS}}{K_{IP}}\right) \times \frac{Pe_i^S}{e^{Pe_i^S} - 1}\right)$$

$$- CLup \times V_i^E \times C_i^P \times F_{up}^E + CLup \times V_i^E \times C_i^E \times F_{rec}^E$$

$$- Pup \times \left(1 - \frac{C_i^M}{K_M + C_i^M}\right) \times V_i^M \times C_i^P \times F_{up}^M + CLup \times V_i^M \times C_i^M \times F_{rec}^M$$

Organ Sub-Compartment: Macrophages

Equation S8: Concentration of NP in Organ Macrophages (i = all organs except the Liver)

$$\frac{dC_i^M}{dt} = Pup \times \left(1 - \frac{C_i^M}{K_M + C_i^M}\right) \times C_i^{IS} - CLup \times C_i^M$$

Equation S9: Concentration of NP in Organ Macrophages in Liver (i = Liver)

$$\frac{dC_i^M}{dt} = Pup \times \left(1 - \frac{C_i^M}{K_M + C_i^M}\right) \times (C_i^P \times F_{up}^M + C_i^{IS} \times (1 - F_{up}^M)) - CLup \times C_i^M$$

Organ Sub-Compartment: Vascular Endothelium

Equation S10: Concentration of NP in Vascular Endothelium

$$\frac{dC_i^E}{dt} = CLup \times (C_i^P \times F_{up}^E + C_i^{IS} \times \left(1 - F_{up}^E\right)) - CLup \times C_i^E$$

Liver Sub-Compartment: Hepatocytes

Equation S11: Concentration of NP in Hepatocytes in Liver

$$\frac{dC_{Liver}^{H}}{dt} = CLup \times C_{Liver}^{IS} - CLup \times C_{Liver}^{H} - K_{Bile} \times C_{Liver}^{H}$$

Organ Sub-Compartment: Interstitial Space

Equation S12: Amount of NP in Interstitial Space of Organs (i = all organs except the Liver, Stomach, Spleen, Pancreas, Small Intestine and Large Intestine)

$$V_i^{IS} \times \frac{dC_i^{IS}}{dt} = \left(J_i^L \times (1 - \sigma_i^L) \times C_i^P + P_i^L \times S_i \times \left(C_i^P - \frac{C_i^{IS}}{K_{IP}} \right) \times \frac{Pe_i^L}{e^{Pe_i^L} - 1} \right.$$

$$\left. + J_i^S \times \left(1 - \sigma_i^S\right) \times C_i^P + P_i^S \times S_i \times \left(C_i^P - \frac{C_i^{IS}}{K_{IP}} \right) \times \frac{Pe_i^S}{e^{Pe_i^S} - 1} \right)$$

$$- \left(1 - \sigma_i^{IS}\right) \times L_i \times C_i^{IS} - CLup \times V_i^E \times C_i^{IS} \times \left(1 - F_{up}^E\right)$$

$$+ CLup \times V_i^E \times C_i^E \times (1 - F_{rec}^E) - Pup \times \left(1 - \frac{C_i^M}{K_M + C_i^M} \right) \times V_i^M \times C_i^{IS}$$

$$+ CLup \times V_i^M \times C_i^M$$

Equation S13: Amount of NP in Interstitial Space of Portal Organs (i = Stomach, Spleen, Pancreas, Small Intestine and Large Intestine)

$$V_i^{IS} \times \frac{dC_i^{IS}}{dt} = K_{Abs} \times A_{IP} \times F_{Portal}^{IP} \times \frac{V_i}{V_{Portal}}$$

$$+ \left(J_i^L \times (1 - \sigma_i^L) \times C_i^P + P_i^L \times S_i \times \left(C_i^P - \frac{C_i^{IS}}{K_{IP}} \right) \times \frac{Pe_i^L}{e^{Pe_i^L} - 1} \right.$$

$$\left. + J_i^S \times \left(1 - \sigma_i^S\right) \times C_i^P + P_i^S \times S_i \times \left(C_i^P - \frac{C_i^{IS}}{K_{IP}} \right) \times \frac{Pe_i^S}{e^{Pe_i^S} - 1} \right)$$

$$- \left(1 - \sigma_i^{IS}\right) \times L_i \times C_i^{IS} - CLup \times V_i^E \times C_i^{IS} \times \left(1 - F_{up}^E\right)$$

$$+ CLup \times V_i^E \times C_i^E \times (1 - F_{rec}^E) - Pup \times \left(1 - \frac{C_i^M}{K_M + C_i^M} \right) \times V_i^M \times C_i^{IS}$$

$$+ CLup \times V_i^M \times C_i^M$$

Equation S14: Amount of NP in the Interstitial Space of Liver (i = Liver)

$$V_i^{IS} \times \frac{dC_i^{IS}}{dt} = \left(J_i^L \times (1 - \sigma_i^L) \times C_i^P + P_i^L \times S_i \times \left(C_i^P - \frac{C_i^{IS}}{K_{IP}}\right) \times \frac{Pe_i^L}{e^{Pe_i^L} - 1}\right.$$

$$\left. + J_i^S \times \left(1 - \sigma_i^S\right) \times C_i^P + P_i^S \times S_i \times \left(C_i^P - \frac{C_i^{IS}}{K_{IP}}\right) \times \frac{Pe_i^S}{e^{Pe_i^S} - 1}\right)$$

$$- \left(1 - \sigma_i^{IS}\right) \times L_i \times C_i^{IS} - CLup \times V_i^E \times C_i^{IS} \times \left(1 - F_{up}^E\right)$$

$$+ CLup \times V_i^E \times C_{Liver}^E \times (1 - F_{rec}^E)$$

$$- Pup \times \left(1 - \frac{C_i^M}{K_M + C_i^M}\right) \times V_i^M \times C_i^{IS} \times (1 - F_{up}^M)$$

$$+ CLup \times V_i^M \times C_i^M \times (1 - F_{rec}^M) - CLup \times V_{Liver}^H \times C_i^{IS}$$

$$+ CLup \times V_{Liver}^H \times C_{Liver}^H$$

Miscellaneous Equations: Pores

Equation S15: Vascular Reflection Coefficient across Large Pores

$$\sigma_i^L = \begin{cases} 1 - \dfrac{(1 - \gamma_i^L)^2 \times [2 - (1 - \gamma_i^L)^2] \times \left(1 - \frac{\gamma_i^L}{3}\right)}{1 - \frac{\gamma_i^L}{3} + \frac{2 \times (\gamma_i^L)^2}{3}} & , \gamma_i^L < 1 \\ 0.999 & , \gamma_i^L \geq 1 \end{cases}$$

Equation S16: Vascular Reflection Coefficient across Small Pores

$$\sigma_i^S = \begin{matrix} 1 - \dfrac{\left(1 - \gamma_i^S\right)^2 \times \left[2 - \left(1 - \gamma_i^S\right)^2\right] \times \left(1 - \frac{\gamma_i^S}{3}\right)}{1 - \frac{\gamma_i^S}{3} + \frac{2 \times \left(\gamma_i^S\right)^2}{3}} & , \gamma_i^S \leq 1 \\ 0.999 & , \gamma_i^S \geq 1 \end{matrix}$$

Equation S17: Permeability across Large Pores

$$P_i^L = \begin{cases} \dfrac{(1 - \gamma_i^L)^{\frac{9}{2}}}{1 - 0.3956 \times \gamma_i^L + 1.0616 \times (\gamma_i^L)^2} \times \dfrac{RT \times \alpha_i^L \times 4 \times Lp_i}{3 \times \pi \times N_a \times r^{NP} \times (r_i^L)^2} & , \gamma_i^L < 1 \\ 1 \cdot 10^{-21} & , \gamma_i^L \geq 1 \end{cases}$$

Equation S18: Permeability across Small Pores

$$P_i^S = \begin{cases} \frac{(1-\gamma_i^S)^{\frac{9}{2}}}{1-0.3956\times\gamma_i^S+1.0616\times\left(\gamma_i^S\right)^2}\times\frac{RT\times(1-\alpha_i^L)\times 4\times Lp_i}{3\times\pi\times N_a\times r^{NP}\times(r_i^S)^2} & ,\gamma_i^S<1 \\ 1\cdot 10^{-21} & ,\gamma_i^S\geq 1 \end{cases}$$

Miscellaneous Equations: Fluid Flux

Equation S19: Lymph Flow Rate

$$L_i = PLQ_i \times F_i^{lymph}$$

Equation S20: Fluid Flux Rate across Large Pores

$$J_i^L = \alpha_i^L \times L_i + F_i^{Jiso} \times (1-\alpha_i^L) \times L_i \times \left(\frac{V_i}{V_{i_{mouse}}}\right)^{-\frac{1}{3}}$$

Equation S21: Fluid Flux Rate across Small Pores

$$J_i^S = (1-\alpha_i^L) \times L_i - F_i^{Jiso} \times (1-\alpha_i^L) \times L_i \times \left(\frac{V_i}{V_{i_{mouse}}}\right)^{-\frac{1}{3}}$$

Miscellaneous Equations: Endothelium

Equation S22: Vascular Endothelial Surface Area

$$S_i = k_e \times F_i^V \times V_i$$

Equation S23: Vascular Endothelial Volume

$$V_i^E = S_i \times d_e$$

Miscellaneous Equations: Intraperitoneal Space

Equation S24: Amount of NP in the Intraperitoneal Space

$$A^{IP} = F \times Ro - K_{Abs} \times A^{IP}$$

Equation S25: Total Volume of Portal Organs

$$V_{Portal} = V_{Stomach} + V_{Spleen} + V_{Pancreas} + V_{SInt} + V_{LInt}$$

Parameter Values

Table S2. Anatomical Values for Mouse.

Parameter	Notation	Value	Unit
Body Mass	BM	28	g
Hematocrit	HCT	0.45	
Plasma Cardiac Output	CO_{Plasma}	240.7	mL/h

Table S3. Organ Volumes and Composition for Mouse.

	V_i (mL)	V_i^P (mL)	V_i^E (mL)	V_i^{IS} (mL)	V_i^C (mL)	V_i^M (mL)	V_i^H (mL)	SA_i cm^2
Heart	0.1266	0.018243	0.000945	0.01266	0.079825	0.001597	-	31.51074
Kidney	0.4531	0.026167	0.001356	0.09062	0.313549	0.006271	-	45.196725
Muscle	13.326	0.190562	0.009875	1.59912	11.37053	0.227411	-	329.1522
Skin	3.865	0.040389	0.002093	1.16723	2.622242	0.052445	-	69.76325
Brain	0.227	0.004619	0.000239	0.000908	0.217454	0.008698	-	7.97905
Adipose	1.33	0.007315	0.000379	0.17955	1.136771	0.045471	-	12.635
Gonads	0.333	0.025641	0.001329	0.022977	0.262074	0.010483	-	44.289
Liver	1.73	0.109423	0.00567	0.28199	1.24339	0.124339	0.9325	189.0025
Stomach	0.1466	0.00258	0.000134	0.01466	0.127115	0.005085	-	4.45664
Spleen	0.133	0.020628	0.001069	0.01995	0.074475	0.022343	-	35.6307
Pancreas	0.173	0.017127	0.000887	0.02076	0.120213	0.004809	-	29.583
S.Intestine	1.874	0.024737	0.001282	0.176156	1.651586	0.066063	-	42.7272
L.Intestine	0.836	0.011035	0.000572	0.078584	0.73678	0.029471	-	19.0608
Bone	2.11	0.047581	0.002466	0.211	1.810024	0.072401	-	82.1845
Lung	0.133	0.045792	0.002373	0.025004	0.022365	0.000895	-	79.0951
Plasma	0.55	-	-	-	-	0.011	-	-
Lymph	0.113	-	-	-	-	0.00452	-	-

Table S4. Fluid Flow Rates for Mouse.

	PLQ_i (mL/h)	L_i (mL/h)	$Jiso_i$ (mL/h)	J_i^L (mL/h)	J_i^S (mL/h)	F_i^{lymph}	F_i^{Jiso}
Heart	12.31365	0.018101	0.015001	0.015906	0.002195	0.00147	0.96
Kidney	57.17012	0.040534	0.026629	0.028655	0.011878	0.000709	0.761
Muscle	40.01798	0.080436	0.020276	0.024298	0.056138	0.00201	0.292
Skin	18.03486	0.063483	0.033813	0.036987	0.026496	0.00352	0.617
Brain	5.728368	0.000416	0.000145	0.000166	0.00025	0.0000727	0.404
Adipose	1.7556	0.013237	0.004082	0.004103	0.008493	0.00754	0.357
Gonads	2.109888	0.02342	0.019412	0.020583	0.002837	0.0111	0.96
Liver	15.36863	0.307373	0.053654	0.299552	0.007821	0.02	0.96
Stomach	4.8378	0.009676	0.008018	0.008502	0.001173	0.002	0.96
Spleen	3.9501	0.078607	0.000143	0.063029	0.015578	0.0199	0.01
Pancreas	2.2836	0.069193	0.000598	0.004057	0.065136	0.0303	0.01
S.Intestine	43.98203	0.085765	0.013252	0.017541	0.068224	0.00195	0.179
L.Intestine	21.99867	0.316781	0.048943	0.064782	0.251999	0.0144	0.179
Bone	11.1408	0.007375	0.006108	0.006477	0.000899	0.000662	0.96
Lung	240.7	0.008562	7.40E-05	0.000502	0.00806	-	0.01
Plasma	240.7	-	-	-	-	-	-
Lymph	-	1.122958	-	-	-	-	-

Table S5. Extravasation Parameters.

	r^L (nm)	r^S (nm)	α_i^L	LP_i (mL/h/N)
Heart	25	4.5	0.05	0.03096
Kidney	25	4.5	0.05	0.27
Muscle	25	4.5	0.05	0.01944
Skin	25	4.5	0.05	0.04206
Brain	25	4.5	0.05	0.000108
Adipose	25	4.5	0.05	0.01944
Gonads	25	4.5	0.05	0.01944
Liver	33	9	0.8	0.084
Stomach	25	4.5	0.05	0.0858
Spleen	33	9	0.8	0.084
Pancreas	25	4.5	0.05	0.0696
S.Intestine	25	4.5	0.05	0.3324
L.Intestine	25	4.5	0.05	0.4038
Bone	25	4.5	0.05	0.01944
Lung	25	4.5	0.05	0.01224

Table S6. Macrophage Content.

	F_i^M
Heart	0.02
Kidney	0.02
Muscle	0.02
Skin	0.02
Brain	0.04
Adipose	0.04
Gonads	0.04
Liver	0.1
Stomach	0.04
Spleen	0.3
Pancreas	0.04
S.Intestine	0.04
L.Intestine	0.04
Bone	0.04
Lung	0.04
Plasma	0.02
Lymph	0.04

Table S7. Model Constants.

Parameter	Notation	Value	Unit
Gas constant at body temperature (37°C)	RT	2.58E+05	N·cm/mol
Proportionality constant for vascular endothelium	k_e	950	cm²/mL
Thickness of vascular endothelium	d_e	3.00E-05	cm
Avogadro's number	N_a	6.02E+23	1/mol
Interstitial:Plasma partition coefficient	K_{IP}	0.96	-
Rate of pinocytosis and exocytosis per mL of endothelium, macrophages and hepatocytes	$CLup$	5.00E-02	L/h/L
Fraction of pinocytic uptake from organ plasma space	F_{up}^{E}	0.5	-
Fraction of exocytosis into organ plasma space	F_{rec}^{E}	0.5	-
Rate of phagocytosis per mL of macrophages	Pup	0.995	L/h/L
Michaelis-Menten constant for saturable phagocytosis	K_M	5000	μg/mL
Fraction of phagocytic uptake from liver plasma	F_{up}^{M}	0.5	-
Fraction of exocytosis from liver macrophages into plasma	F_{rec}^{M}	0.5	-
Lymphatic reflection coefficient	σ^{IS}	0.64	-
Rate of excretion into bile from liver hepatocytes	K_{Bile}	0.0128	1/h
Intraperitoneal bioavailability	F	0.76	-
Fraction absorbed into the portal system	F_{Portal}	0.9	-
Rate of absorption from the intraperitoneal space	K_{Abs}	0.05	1/h

Table S8. Anatomical Values for Rat.

Parameter	Notation	Value	Unit
Body Mass	BM	280	g
Hematocrit	HCT	0.45	
Plasma Cardiac Output	CO_{Plasma}	1747.762	mL/h

Table S9. Organ Volumes and Composition for Rat.

	V_i (mL)	V_i^P (mL)	V_i^E (mL)	V_i^{IS} (mL)	V_i^C (mL)	V_i^M (mL)	V_i^H (mL)	SA_i cm²
Heart	0.983	0.14165	0.00734	0.0983	0.619814	0.012396	-	244.6687
Kidney	2.827	0.163259	0.00846	0.5654	1.956305	0.039126	-	281.9933
Muscle	149.97	2.144571	0.111128	17.9964	127.9633	2.559265	-	3704.259
Skin	49.17	0.513827	0.026626	14.84934	33.3598	0.667196	-	887.5185
Brain	2.089	0.042511	0.002203	0.008356	2.001148	0.080046	-	73.42835
Adipose	12.292	0.067606	0.003503	1.65942	10.50616	0.420246	-	116.774
Gonads	3.073	0.236621	0.012261	0.212037	2.418482	0.096739	-	408.709
Liver	12.6615	0.80084	0.041498	2.063825	9.100105	0.91001	6.825079	1383.269
Stomach	1.352	0.023795	0.001233	0.1352	1.172303	0.046892	-	41.1008
Spleen	0.73756	0.114396	0.005928	0.110634	0.413006	0.123902	-	197.5923
Pancreas	1.598	0.158202	0.008198	0.19176	1.110402	0.044416	-	273.258
S.Intestine	6.146	0.081127	0.004204	0.577724	5.416568	0.216663	-	140.1288
L.Intestine	2.676	0.035323	0.00183	0.251544	2.358402	0.094336	-	61.0128
Bone	19.422	0.437966	0.022695	1.9422	16.6608	0.666432	-	756.4869
Lung	1.229	0.423145	0.021927	0.231052	0.206667	0.008267	-	730.8863
Plasma	6.56	-	-	-	-	0.1312	-	-
Lymph	1.15	-	-	-	-	0.046	-	-

Table S10. Fluid Flow Rates for Rat.

	PLQ_i (mL/h)	L_i (mL/h)	$Jiso_i$ (mL/h)	J_i^L (mL/h)	J_i^S (mL/h)	F_i^{lymph}	F_i^{Jiso}
Heart	161.7344	0.23775	0.099503	0.111391	0.126359	0.00147	0.96
Kidney	374.5167	0.265532	0.094757	0.108033	0.157499	0.000709	0.761
Muscle	304.3641	0.611772	0.068817	0.099405	0.512366	0.00201	0.292
Skin	236.5765	0.832749	0.190001	0.231638	0.601111	0.00352	0.617
Brain	54.06729	0.003931	0.000654	0.00085	0.00308	0.0000727	0.404
Adipose	16.22544	0.12234	0.017978	0.018175	0.098244	0.00754	0.357
Gonads	19.47053	0.216123	0.085406	0.096212	0.119911	0.0111	0.96
Liver	81.14249	1.62285	0.145903	1.444183	0.178666	0.02	0.96
Stomach	45.96786	0.091936	0.036332	0.040928	0.051007	0.002	0.96
Spleen	25.69276	0.511286	0.000525	0.409554	0.101732	0.0199	0.01
Pancreas	20.95649	0.634982	0.002614	0.034363	0.600619	0.0303	0.01
S.Intestine	101.409	0.197748	0.020566	0.030453	0.167294	0.00195	0.179
L.Intestine	202.817	2.920565	0.306176	0.452204	2.468361	0.0144	0.179
Bone	102.7597	0.068027	0.026882	0.030283	0.037743	0.000662	0.96
Lung	1747.762	0.062118	0.000256	0.003362	0.058756	-	0.01
Plasma	1747.762	-	-	-	-	-	-
Lymph	-	8.399707	-	-	-	-	-

Model Building PK: This Study

Particles: EGCG and Curcumin-capped Gold Nanoparticles

Hydrodynamic Radius: 12 nm

Dose: 10 mg Au/kg IP

Animal: Mouse

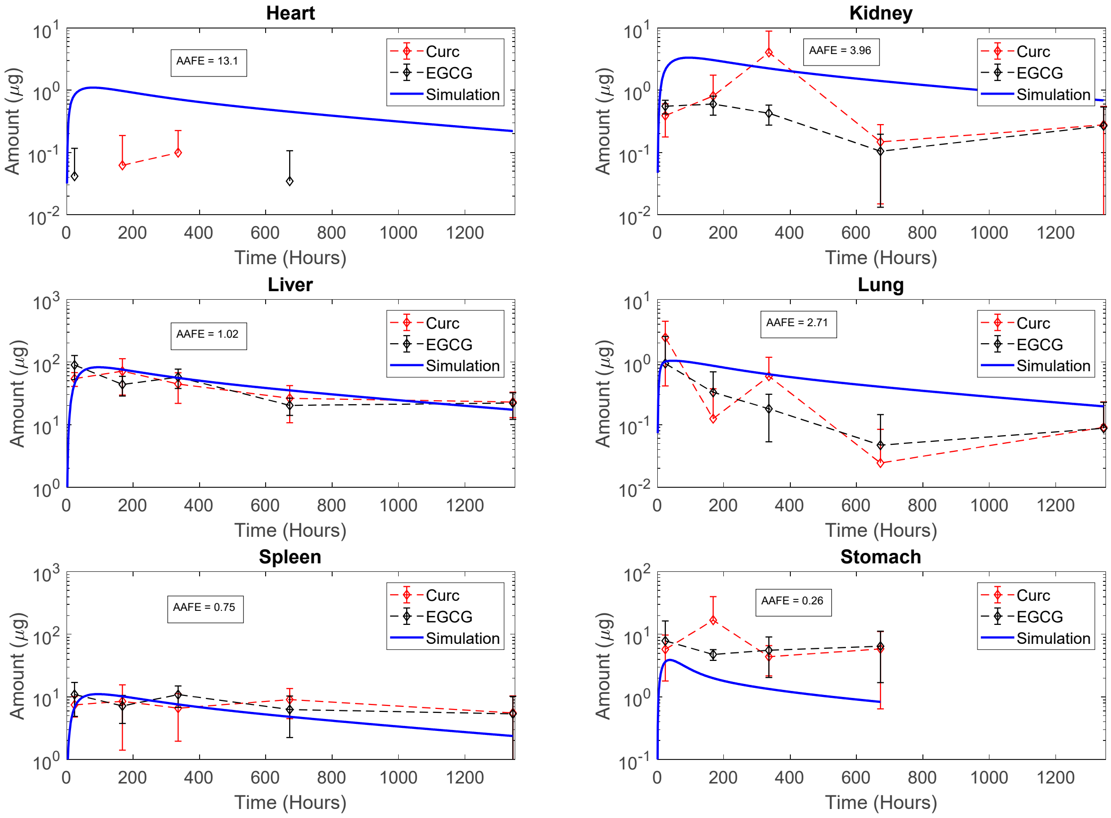

Figure S1. Comparison of Model Simulations to Mouse Biodistribution Data from this Study.

Model Evaluation PK: Balasubramanian et al.

Particles: Citrate-capped Gold Nanoparticles

Hydrodynamic Radius: 30 nm

Dose: 0.01 mg Au/kg IV

Animal: Rat

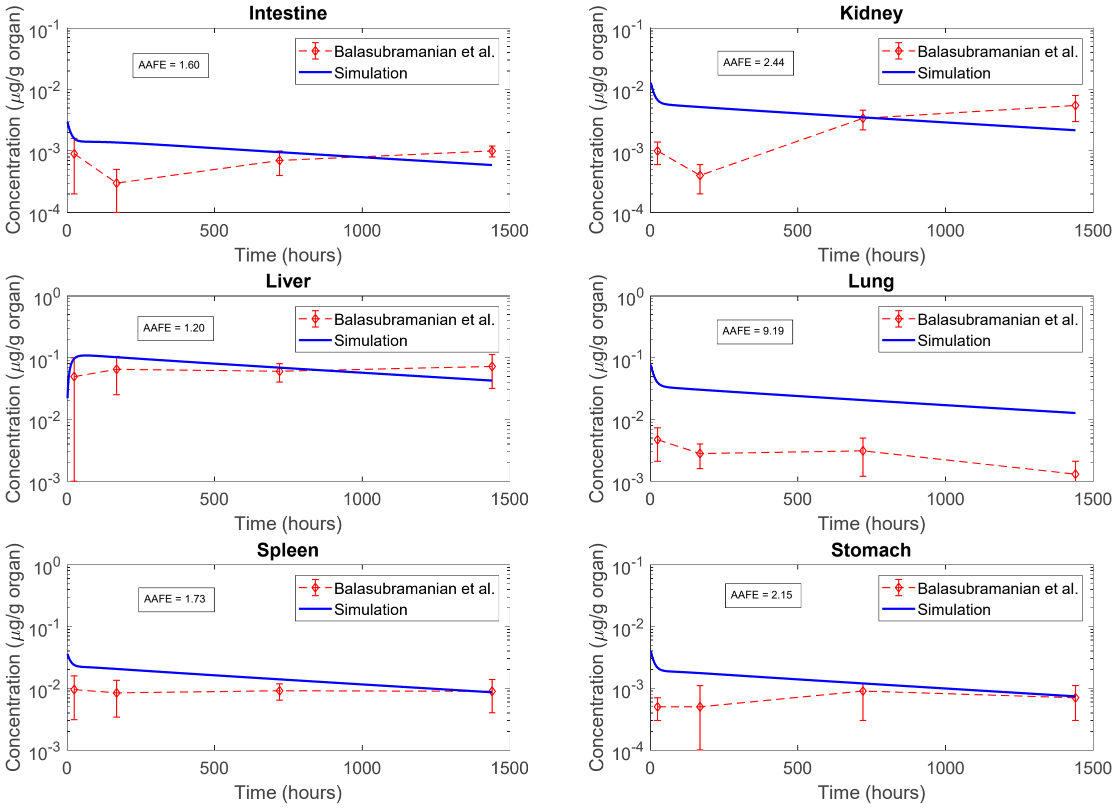

Figure 2. Comparison of Model Simulations to Rat Biodistribution Data from Balasubramanian et al.

Model Evaluation PK: Fraga et al.

Particles: Citrate-capped Gold Nanoparticles

Hydrodynamic Radius: 23 nm

Dose: 0.7 mg Au/kg IV

Animal: Rat

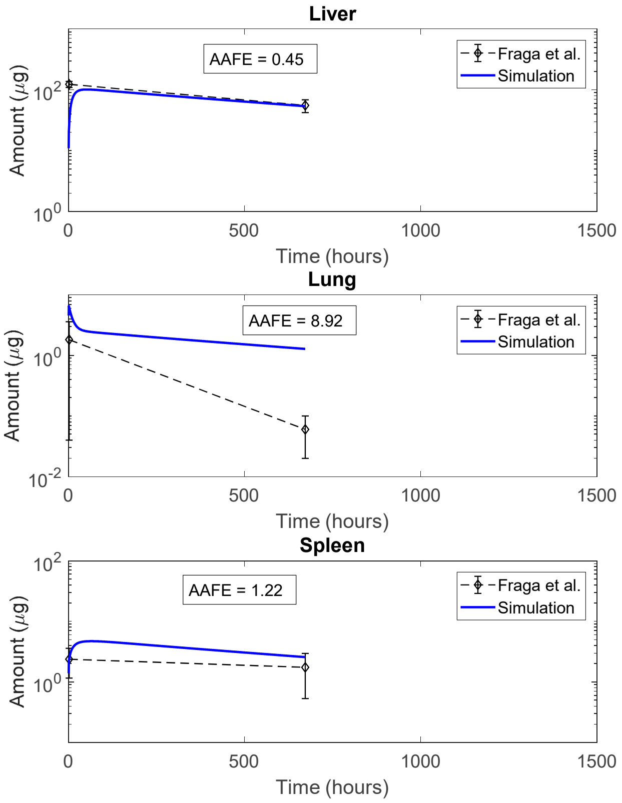

Figure S3. Comparison of Model Simulations to Rat Biodistribution Data from Fraga et al.
